# The Potential Impact of Animal Science Research on Global Maternal and Child Nutrition and Health: A Landscape Review^1^,^2^

**DOI:** 10.3945/an.116.013896

**Published:** 2017-03-10

**Authors:** Jack Odle, Sheila K Jacobi, R Dean Boyd, Dale E Bauman, Russell V Anthony, Fuller W Bazer, Adam L Lock, Andrew C Serazin

**Affiliations:** 3 Laboratory of Developmental Nutrition, Department of Animal Science, North Carolina State University, Raleigh, NC;; 4 Department of Animal Science, The Ohio State University, Wooster, OH;; 5 Department of Science Integration, The Hanor Company, Spring Green, WI;; 6 Department of Animal Science, Cornell University, Ithaca, NY;; 7 Animal Reproduction and Biotechnology Laboratory, Department of Biomedical Sciences, Colorado State University, Fort Collins, CO;; 8 Department of Animal Science, Texas A&M University, College Station, TX;; 9 Department of Animal Science, Michigan State University, East Lansing, MI; and; 10 Templeton World Charity Foundation, Nassau, Bahamas

**Keywords:** global health, growth stunting, animal models, pregnancy, lactation, fatty acids, amino acids, zinc, iron, phytase

## Abstract

High among the challenges facing mankind as the world population rapidly expands toward 9 billion people by 2050 is the technological development and implementation of sustainable agriculture and food systems to supply abundant and wholesome nutrition. In many low-income societies, women and children are the most vulnerable to food insecurity, and it is unequivocal that quality nutrition during the first 1000 d of life postconception can be transformative in establishing a robust, lifelong developmental trajectory. With the desire to catalyze disruptive advancements in global maternal and child health, this landscape review was commissioned by the Bill & Melinda Gates Foundation to examine the nutritional and managerial practices used within the food-animal agricultural system that may have relevance to the challenges faced by global human health. The landscape was categorized into a framework spanning *1*) preconception, *2*) gestation and pregnancy, *3*) lactation and suckling, and *4*) postweaning and toddler phases. Twelve key findings are outlined, wherein research within the discipline of animal sciences stands to inform the global health community and in some cases identifies gaps in knowledge in which further research is merited. Notable among the findings were *1*) the quantitative importance of essential fatty acid and amino acid nutrition in reproductive health, *2*) the suggested application of the ideal protein concept for improving the amino acid nutrition of mothers and children, *3*) the prospect of using dietary phytase to improve the bioavailability of trace minerals in plant and vegetable-based diets, and *4*) nutritional interventions to mitigate environmental enteropathy. The desired outcome of this review was to identify potential interventions that may be worthy of consideration. Better appreciation of the close linkage between human health, medicine, and agriculture will identify opportunities that will enable faster and more efficient innovations in global maternal and child health.

## Introduction

There is nothing as important to the advancement of global maternal and infant health as the maintenance of healthy pregnancies, the reduction of maternal complications in pregnancy and childbirth, and the promotion of optimal postnatal health and development of children. As the 2013 Lancet *Series on Maternal and Child Nutrition* (1) makes clear, despite progress in national commitment, increases in donor funding, and engagement of civil society and the private sector, “improvements in nutrition still represent a massive unfinished agenda.” The same can be argued for other key metrics of maternal and child health, including maternal mortality and morbidity, still births, preterm births, neonatal survival, and childhood infectious diseases.

The optimization of reproductive success for the mother and offspring have long been at the center of farm animal husbandry. In the United States, the >150 y history of the land-grant university system (2) brought great focus to the sciences undergirding farm animal production, including nutrition, reproduction, genetics, growth biology, lactation, and health. Indeed, livestock farms can be viewed as systems that manage the complex variables of environment, nutrition, genetics, pathogen exposure, and application of health interventions to achieve optimal animal productivity and growth in a highly controlled manner. Modern farms demonstrate successful management of these variables on a large scale. Consider nutrition, for example: pig farms routinely formulate >10 diets to meet the animals’ unique physiologic nutrient requirements across the life cycle. In many cases, animals that develop illness are segregated into settings in which they are fed enhanced therapeutic diets. In this way, animal nutritionists optimize diets for ∼50 essential nutrients at best cost to feed tens of thousands of animals in their care every day. The practice of precision nutrition has led to substantial gains in both the efficiency and productivity of farms and decreased environmental impact, as well as a reduction in common diseases during periods of stress. By comparison, the data available to human nutritionists for the optimization of diets in these highly critical windows of human development are sorely lacking.

There are structural factors that favor the process of innovation in the animal sciences. Trials aimed at developing new products are less costly, confounding factors such as genetic background and environmental variables can be controlled, and adoption of successful innovations is fueled by market incentives. By contrast, human societies, lifestyles, and diets are much more complex than those of animals and less subject to experimentation or standardization. Ethical concerns also preclude the use of invasive experimental approaches and interventions that are common practice in animal sciences. Given the high degree of underlying biological commonality within mammals, much insight can be gained through extrapolation from agriculture species, termed dual-benefit, “agrimedical” application and “one health” (3).

Despite this shared biology across species and overlapping challenges that mothers and their offspring face, the disconnection between the animal science research community and the human global health research community is large, with little cross-sharing of knowledge. The division between these corresponding research communities underscores the need to bridge knowledge across the disciplines. Indeed, there is opportunity to accelerate progress by better use of existing knowledge across these parallel yet divergent fields.

**FIGURE 1:**
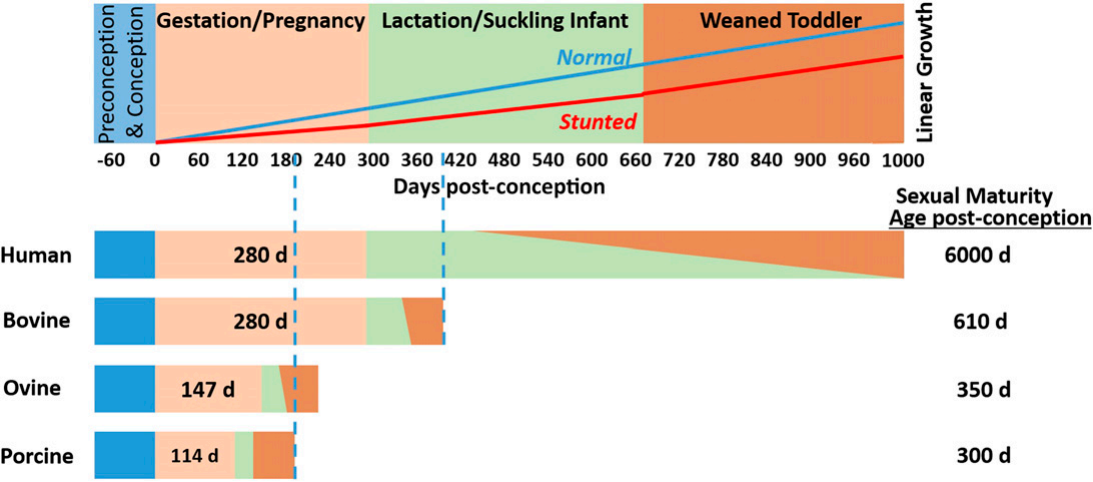
Landscape review framework. Four phases of human early-life growth and development (upper panel) are scaled against the accelerated development of agricultural species (lower bars) based on age at sexual maturity. Normal and stunted linear growth over the first 1000 d postconception are illustrated. This review categorically addresses opportunities for improving maternal and infant health and growth during these 4 developmental phases, drawing upon insights gleaned from research on agricultural species.

For these reasons, this landscape review highlights knowledge and advances in the animal sciences research community for consideration by the human global health community. The review is framed according to a chronologic, developmental approach (**Figure 1**), identifying opportunities during the following physiological windows of the life cycle for humans and animals: *1*) preconception, *2*) gestation and pregnancy, *3*) lactation and suckling, and *4*) postweaning and toddler.

The critical importance of the first 1000 d in establishing a developmental trajectory for healthy infants is well recognized (4), and the dire consequences of poor nutrition and stunting during this time have lifelong consequences such that early, progressive interventions are essential. When compared with agricultural species that display starkly greater growth rates (e.g., piglets grow ∼10 times faster than do human infants), the relative nutrient requirements and developmental timelines are altered accordingly. For example, when scaled according to age at sexual maturity (Figure 1), 1000 d of human development corresponds to ∼400 d for cattle and 180 d for pigs, with sheep being intermediate. Scaling for body size is another important factor affecting metabolic rate and nutrient requirements (5), as is the precocial neurologic development of livestock species (6). Furthermore, although gestation length is similar for humans and bovine species (280 d), that of ovine and porcine species is ≤50% in duration (140 and 114 d, respectively). Another large difference between species is the abrupt and early weaning of pigs and dairy calves compared with the more gradual weaning typically occurring in humans, beef cattle, and sheep (Figure 1).

Among livestock species, the ovine model has been used extensively for studies of prenatal development, and the dairy bovine model extensively for studies of milk and lactation. Because the porcine species is similar to humans as the only omnivorous, monogastric species among those considered, this review draws heavily upon insights from that species across all developmental phases. Because it is the only litter-bearing species considered, extrapolation from the porcine species to humans bears special attention. Another constraint on extrapolation from farm animal species is that animals are harvested for the human food chain at relatively young ages (e.g., 6 and 18 mo for porcine and bovine species, respectively). Therefore, the long-term health impacts of early-life interventions can only be assessed with animals maintained for herd reproduction, and these animals also have relatively short lifespans compared with those of humans.

As each developmental phase is reviewed, in some cases specific interventions are described that have been broadly adopted by the livestock industry and provide quantifiable evidence of impact on relevant health indicators. In other cases, proposed gaps in knowledge are identified that currently impede progress. The review canvasses multiple examples to provide concrete illustrations in which application of insights from animal science could represent practical starting points for innovations in human maternal and child health. The intent was not to be prescriptive, but rather to identify opportunities that may be relevant for present-day global health and nutrition practices.

## Current Status of Knowledge: Findings from Animal Science Research with the Potential to Improve Global Maternal and Child Nutrition and Health

Healthy reproduction perpetuates the species, but the physiologic well-being of progeny is determined by the internal environment that the mother provides, that environment being influenced by the preconception health and physiologic status of the mother-to-be. For example, extremes in nutrition (over or under) and environmental temperature can markedly affect conception rates and fetal development, and epigenetic effects that can span multiple generations can manifest in the developing embryo and fetus (7).

Outcomes of fetal programming have been studied epidemiologically in humans, but specific cause and effect changes have been largely determined with the use of murine, nonhuman primate, porcine, and ovine models. The ovine species has proven especially informative because metabolic and endocrine changes better reflect humans than do data from murine species. Because of their larger size, the effects induced by environmental stressors can be studied throughout pregnancy with the use of indwelling catheters to collect serial blood samples from the ewe and her fetuses (8, 9).

Information arising from ovine and porcine models provides important details about outcomes arising from extreme heat and malnutrition. Because these perturbations can produce lasting effects that are not easily reversed, interventions must occur early (preconception and early pregnancy). Furthermore, the adequate intake of dietary essential FAs (EFAs)^11^ by gestating and lactating dams improves reproductive outcomes. These findings represent solutions to key gaps in knowledge that may be useful interventions in human reproduction, as well as in high-, middle-, and low-income countries.

### Preconception nutrition is linked to offspring viability and health

#### Key finding 1. Ovine research has shown that preconception is a critical period in which nutritional insult has adverse consequences for offspring.

The sheep is an excellent model to study the impacts of maternal over- or undernutrition on offspring, because it exhibits many similarities with humans (10). Both species produce well-developed precocial offspring; both exhibit similar newborn-to-maternal weight ratios; both display similar temporal patterns of fetal tissue and organ development; and both rely on glucose as a predominant fetal energy source.

Preconception nutritional status typically does not affect birth weight in the ovine model, unless deviations in feed consumption are extreme, but it does affect postnatal and adult phenotypes (11). Both under- and overnutrition of prospective mothers set a trajectory in offspring toward increased adiposity, hyperphagia and dysfunction of insulin and glucose responsiveness, and homeostasis. These outcomes closely track those outlined by Barker (12) in human development.

Obesity at conception and excess pregnancy weight gain elevate the risk of adverse health consequences in human offspring (13). Studies with sheep show that diet intervention in early pregnancy can largely overcome prepregnancy obesity (14). Ovine mothers that were over- or underfed before conception (resulting in obese or malnourished states) led to offspring that each had deleterious metabolic phenotypes. The progeny of ewes that experienced long-term preconception overnutrition had 33% more body fat at 4 mo postbirth. However, body fat was decreased by ∼50% if the overfed prospective mothers were restricted to a diet that was only slightly above maintenance level for 30 d before conception. Intervention during early pregnancy might be beneficial. When overfed, obese ewes were restricted to normal energy intake beginning in early pregnancy (day 28), deleterious alterations in fetal growth, adiposity, and glucose and insulin metabolism were prevented. This important finding suggests that moderate diet modification during preconception and early pregnancy can alter the intrauterine environment enough to produce substantial change in health outcomes for the progeny.

#### Key finding 2. Bovine research has revealed specific biological effects behind the compromised ability to achieve and maintain pregnancy, if extreme energy malnutrition occurs preconception.

The impact of preconception malnutrition, specifically the negative energy balance that occurs naturally during the dairy lactation cycle, has been studied extensively because of a progressive decline in the fertility of dairy cows observed over the past few decades. The impact on oocyte and embryo quality is compromised in relation to the severity of energy malnutrition (15). The relation between energy malnutrition and specific biological effects on ova and embryo development is a new discovery. The practical outcome is failure of the cow to conceive, or if the cow conceives, failure to maintain pregnancy.

#### Key finding 3. Porcine research has quantified the amount of EFAs required preconception to increase the probability of conception and to retain pregnancy.

**FIGURE 2:**
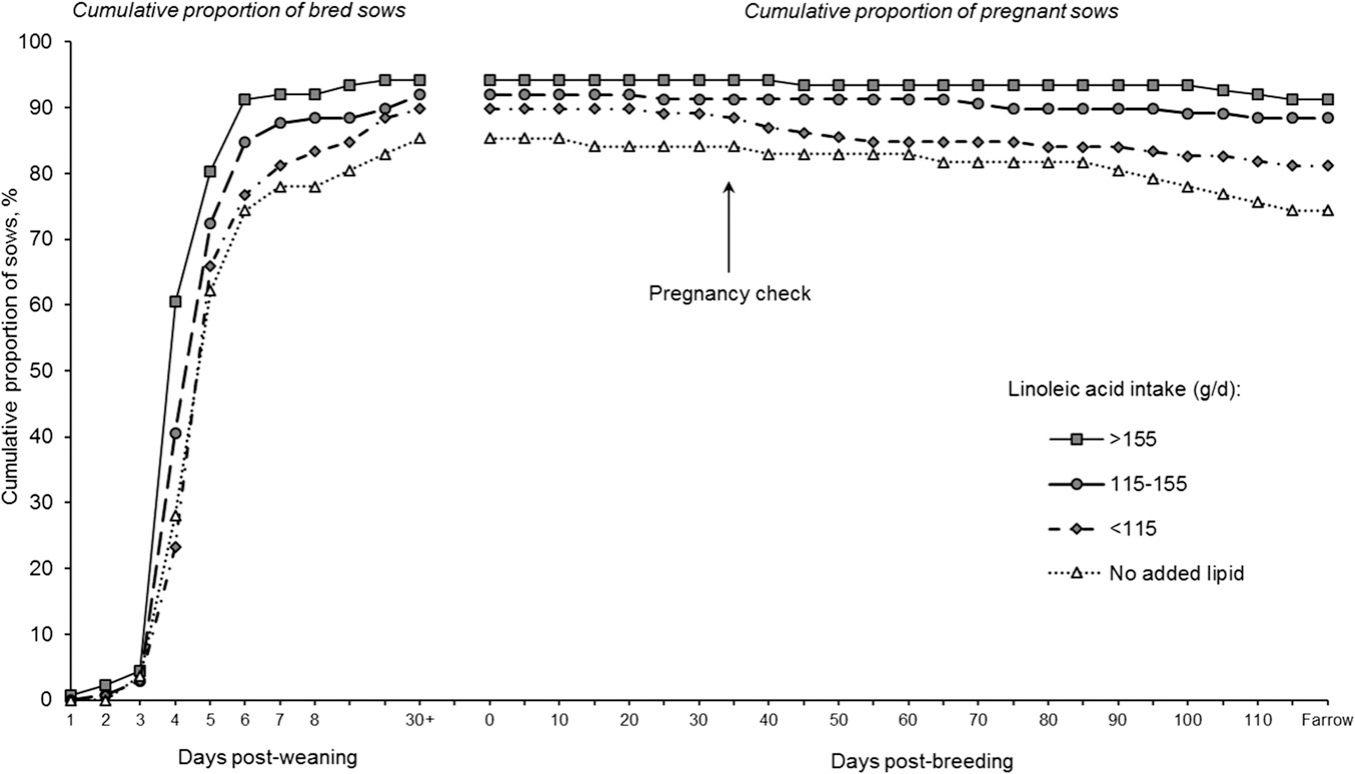
Increasing linoleic acid intake during lactation progressively improves the subsequent reproductive cycle of sows by >15%. Data represent the cumulative proportion of bred and pregnant sows relative to the number that weaned a litter. Sows fed diets containing no added lipids consumed a mean ± SE of 84.4 ± 20.3 g/d of linoleic acid. Values are means derived from *n* = 543 mature sows producing their third, fourth, or fifth litters. Reproduced from reference 17 with permission.

Food animal research has shown that energy considerations, important to reproduction, involve *1*) the amount of energy consumed in general, and *2*) specific EFAs that are energy yielding but, more importantly, are substrates for a plethora of potent bioactive signaling molecules. Although EFAs are important in reproduction for both animals and humans, recent research by Rosero and coworkers (16, 17) defined requirements (grams per day), for the first time in any species, to our knowledge, for both linoleic acid (18:2n–6) and α-linolenic acid (18:3n–3) in reproducing swine. They showed that dietary EFAs affected not only the ability to express estrus and conceive, but also to maintain pregnancy (**Figure 2**). Although it appeared that maximal conception and pregnancy maintenance could be supported by either EFA separately, best outcomes were achieved with a minimum of 125 g linoleate/d and 10 g α-linolenate/d (∼3.30% and 0.26% of as-fed diet, respectively). A further observation was that the number of progeny born per sow increased as linoleate intake increased. These studies were carried out under conditions of heat stress and lactation, when energy balance and EFA status are often low. This model of EFA depletion of otherwise well-nourished sows produced remarkable findings that have implications for women in high-, middle-, and low-income countries. Although EFA biochemistry and physiology has been studied in a number of species, especially the dairy cow (18), quantification of the minimal amount required to maintain conception is novel and obligatory for the fundamental knowledge to be of practical value. Furthermore, the observation that some sows conceived, but could not maintain pregnancy, is important to animal agriculture as well as humans.

In a classic paper, Ness (19) presented the “pregnancy failure hypothesis for women,” a hypothesis that pregnancy failure is in part caused by immune hyper-responsiveness. Pigs also exhibit pregnancy failure, especially under extreme heat stress, and immune hyper-responsiveness is likewise a concern. Accordingly, the pro- and anti-inflammatory properties of n–6 and n–3 EFAs may be important for both animals and for any human populations in which energy intake and/or EFA intake may be deficient. We acknowledge the current use of long-chain n–6 (arachidonate; 20:4n–6) and n–3 [EPA (20:5n–3) and DHA (22:6n–3)] FA supplements for humans, but the application in animal science involved the parent 18-carbon EFA because some downstream intermediates may be essential to enhanced reproductive function. The research by Rosero et al. (16) filled a knowledge gap for achieving and maintaining pregnancy. It does not, however, address the quantitative requirements for EFAs necessary for proper neurological development in the fetus.

##### Global health perspective.

The plane of maternal nutrition before conception is an important determinant of infant phenotype. Over- or undernutrition before conception adversely affects progeny adiposity as well as glucose and insulin and lipid metabolism through adolescence. Animal models suggest that intervention may be beneficial if energy restriction occurs early in pregnancy. The role of EFAs merits consideration in human pregnancy failure. Recommendations for EFA intake are not currently part of dietary guidelines for prospective mothers or pregnant women in most countries, and knowledge gaps are worthy of examination.

### Variables affecting pregnant females that impair offspring growth and health

#### Key finding 4. Ovine and porcine research has illustrated that prolonged hyperthermia during pregnancy increases adiposity and decreases protein deposition in offspring, and this cannot be reversed by rearing progeny without heat stress.

Maternal hyperthermia during pregnancy is a problem in many parts of the world. Domestic animal research suggests that there are large repercussions, if the stress is severe and prolonged. Two recent papers, one reviewing the ovine model (20) and the other a porcine model (21), examined hyperthermic stress during pregnancy to study the effects on body composition and rate of progeny growth.

Zhang et al. (20) reported that when ovine maternal hyperthermia (diurnal 40°C/12 h followed by 35°C/12 h) was imposed from 40 to 120 d of pregnancy (increasing core temperature from 39°C to 40°C), placental and fetal weights were predictably decreased by 55–65% and 45–55%, respectively. Umbilical vein uptake of glucose, amino acids, and oxygen were reduced (50–60%), and fetal insulin and insulin-like growth factor (IGF) I were likewise reduced. Reductions in fetal pancreatic β cell mass and insulin production were coupled to increased catecholamines and decreased amino acids (including leucine) in fetal blood. The key finding was that fetal growth restriction was asymmetric in that skeletal muscle and fetal liver growth and function were disproportionately impaired (20).

Johnson et al. (21) demonstrated that porcine maternal hyperthermia during pregnancy altered postnatal tissue growth. This programming effect resulted in the same postnatal outcome whether offspring were reared through adolescence under thermo-neutral or under heat-stress conditions. Pregnant sows were subjected to a diurnal thermal environment (37°C, daytime; 27°C, nighttime). Offspring born to dams experiencing heat stress during pregnancy (intrauterine heat stress) grew more slowly to 80 kg body weight and were fatter than progeny from pregnant sows reared under thermo-neutral conditions. Independently of postnatal environment, and under conditions of amino acid and energy adequacy, the ratio of lipid-to-protein accretion rate for offspring experiencing intrauterine heat stress was 95% greater than for thermo-neutral controls. There is no known means to counteract the effects of prenatal thermal insult on subsequent growth trajectory in farm animals.

##### Global health perspective.

Heat stress could represent a problem for infants of pregnant women living in hot environments. Animal science research shows that maternal hyperthermia produces growth inhibition and increased body fat composition in offspring, even if they are subsequently reared under thermo-neutral conditions. An intervention is not apparent, except to counter heat stress through physical cooling.

#### Key finding 5. Porcine research has provided structure for pregnancy-stage nutrition priorities to optimally support subsequent lactation and fetal and infant growth.

Boyd et al. (22) described 3 stages of prenatal nutrition for swine that require specific attention, especially for the first-time pregnant sow, because she is smaller and thereby most vulnerable to nutritional insult. Trimester length is longer for the human than for the pig. Length of pregnancy in the pig is 16.5 wk, with each physiologic phase being just under 6 wk in length. Accordingly, the specificity of macronutrient requirements involves *1*) needs for embryonic survival (initial one-third of pregnancy), *2*) needs for maternal body mass growth that are balanced for protein and lipid accumulation (middle one-third of pregnancy), and *3*) needs for the period of rapid fetal and mammary growth (last one-third of pregnancy), with mammary growth continuing after birth. If energy and/or amino acid nutrition is not adequate during the final period, mammary growth may be compromised or it may occur at the expense of body lipid and protein mass (22). Either way, milk output could be compromised in young sows, which would reduce progeny growth.

Energy and essential amino acids are quantitatively the most important, and requirements for these are accurately known for domestic animals, especially reproducing swine. Computation of energy need is straight forward, but estimation of amino acid requirements is more difficult. Energy deficit is readily avoided because accurate prediction equations have been developed to estimate requirements beyond maintenance for conceptus and mammary growth by stage of pregnancy (23). Nonetheless, Noblet et al. (24) demonstrated that a 33% deficit in maintenance energy intake during pregnancy of sows did not impact the number of viable fetuses, but resulted in litters that were 22% lighter. Similarly, a deficit in protein intake during pregnancy resulted in decreased placental weight (21%), and this resulted in an 8% reduction in litter birth weights (25, 26). The extent to which a protein deficit restricts fetal growth depends on the extent to which the protein is able to meet the requirement for all essential amino acids (discussed below).

From a global health perspective, unless adequate energy and quality protein intake (containing adequate essential amino acids) are provided to young, first-time (e.g., teenage) mothers in late pregnancy continuing through lactation, then small maternal body mass (from which to draw energy and amino acids) and mammary growth will be inadequate to support optimal milk production. Decreased maternal body mass and decreased milk secretion could limit postnatal infant growth and may complicate the plight of infants born to young mothers who presumably (as with dairy cows and pigs) may also provide less immune protection.

#### Key finding 6. Porcine research on amino acid nutrition exposes a knowledge gap in human nutrition with respect to meeting the needs of third-trimester–pregnant and lactating mothers.

Perhaps the most urgent gap identified is for amino acid nutrition in late-pregnant and lactating mothers. There is insufficient knowledge of specific amino acid needs for humans to know whether the requirements for all amino acids are met in late-pregnant and lactating mothers and perhaps infants, extending through early childhood and into adolescence (compare WHO, 2007 to NRC swine model, 2012) (23, 27). In contrast to the extensive data available on swine amino acid requirements throughout the life cycle (**Text Box 1**), practical human amino acid nutrition knowledge is sorely lacking. Based on calculations extrapolated from swine amino acid requirements (23), we estimate that multiple amino acids would be severely deficient in late-pregnant young (small) human women sustained on a corn meal diet alone (assuming an intake of 680 g/d for a 50-kg mother). The information needed to conduct this computation is comprehensive and is in routine use for predicting the needs of pregnant pigs and matching them with combinations of feedstuffs to meet the requirement (23). This format and the quantitative relations can be used to predict whether certain foods (or combinations), consumed in known amounts, meet human productive needs.

Text Box 1 **Ideal protein**

Perhaps the greatest advancement in establishing amino acid requirements for domestic animals in the past 25 y has been the implementation of the ideal protein concept (32, 33). This concept has been widely applied in swine, poultry, and dairy cattle nutrition. The computations account for differences in specific amino acid patterns required for various physiologic processes (e.g., maintenance, growth, pregnancy, and lactation). In swine, for example, the requirement for each amino acid is then expressed as a ratio of the requirement for lysine, the first limiting amino acid in typical pig diets. This pattern of essential amino acids is referred to as “ideal protein,” because it would precisely meet the physiologic needs of the animal. Importantly, the ideal amino acid pattern required for maintenance differs from that for growth (32). Thus, it is not surprising that the ideal pattern of amino acids required in early pregnancy and midpregnancy (low tissue deposition) differs from that in late pregnancy (increasing proportion of tissue growth beyond maintenance) (23, 34). The value is illustrated for pregnant sows, in which the requirements for lysine and threonine have been studied, but information is insufficient for the other 8 essential amino acids. The ideal pattern for empirically undetermined amino acids was estimated in pigs based on the mass and composition of tissues and fluids at various stages of pregnancy (34). This approach provides a basis for estimating requirements for all amino acids, without empirically determining the requirement for each individual amino acid (23).

##### Global health perspective.

Requirements for the first 4–5 most-limiting amino acids must be known when formulating maternal and infant diet plans to ensure that needs are met for all essential amino acids. A deficit of ≥1 of these amino acids in late-pregnant mothers compromises nitrogen accretion (28), which limits protein deposition in conceptus, mammary gland, and maternal reserves. Constraint of the latter can also place lactation at risk for young mothers (22) if insufficient labile protein is available to be mobilized in early lactation. Accordingly, late fetal and nursing-infant growth can be stunted.

Animals and humans have requirements for essential amino acids, rather than protein. In practice, human nutrition generally speaks of the ability of a given protein to meet needs because individual amino acid requirements are not well known (27, 29). Some ingredients are so devoid of ≥1 critical amino acids (e.g., tryptophan in corn) that lean gain would be compromised unless properly fortified. Low intake of essential amino acids (because of overall low protein intake and/or intake of proteins with poor amino acid balance) cannot be completely offset by the slow growth of humans relative to that of pigs, because relative maternal, conceptus, and mammary growth in late-pregnant sows (0.435%/d) is not too disparate from humans (i.e., 100–145 g/d in late pregnancy = 0.290%/d).

**FIGURE 3:**
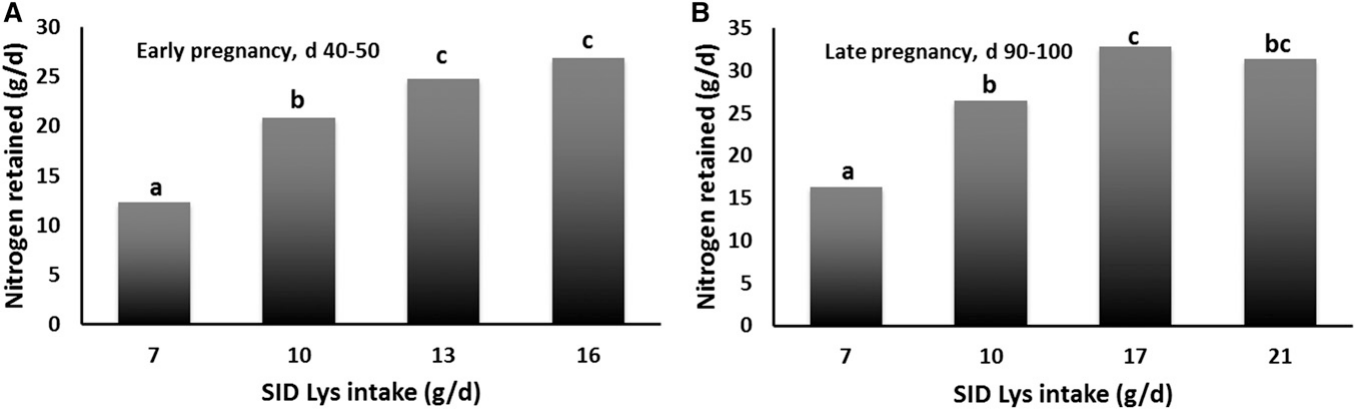
Nitrogen retention by pregnant sows in early (A) and late (B) gestation, fed diets with increasing concentrations of lysine. Values are means; bars without a common letter differ, *P* < 0.05. Linear and quadratic effects, *P* < 0.001. SID, standardized ileal digestible. Adapted from references 30 and 31 with permission.

In young, small, pregnant sows, nitrogen deposition was constrained by >50% (30, 31) when the sows were fed diets containing corn and moderate amounts of soy (5.75–14.25%; as-fed basis) (**Figure 3**). Srichana (28) could not discern between nitrogen deposition for conceptus and maternal protein deposition; however, nitrogen deposition as protein was constrained, and this might reduce the maternal labile protein reserve in early lactation when dietary intake is not adequate (i.e., energy and amino acid outputs exceed diet intake). Diet adequacy is easily demonstrable with the use of the nitrogen retention assay, and it should be known for pregnant and lactating human mothers.

In practice, amino acid adequacy cannot be computed for late-pregnant females without knowing *1*) the net tissue requirement to maintain the mother and to support the conceptus, mammary growth, and a minimum body protein pool size; *2*) the digestibility of ingredient amino acids; and *3*) how efficiently the absorbed amino acids are used for tissue deposition (metabolic efficiency) as maternal, conceptus, and mammary protein. For example, corn containing 7.2% crude protein (as-fed basis) has a total lysine content of 0.215%, but 26% of this is not available [standardized ileal digestible (SID) = 0.74]. Inaccessibility ranges from 13% to 26% for the essential amino acids. Examples of data for the total compared with SID content of 4 selected amino acids in corn are shown in **Table 1** (23). Once absorbed, <100% of any given amino acid is used for protein deposition. For example, ∼65% of absorbed lysine is used for protein deposition in growing pigs or for maternal body protein deposition in sows (23). However, the NRC (23) estimates that only 49% of absorbed lysine is used for conceptus and mammary growth in pigs.

**TABLE 1:** Relative error of computing amino acid contributions for growing pigs from diet ingredients on a total compared with SID basis, with the use of corn as an example^1^

				Difference (total − SID)		
Selected amino acids	Total, %	SID coefficient^2^	SID basis, %	Total − SID, %	Overestimate, g/d	Overestimate, %
Lys	0.215	0.74	0.159	0.056	0.17	35
Ile	0.230	0.82	0.189	0.041	0.12	21
Thr	0.238	0.77	0.183	0.055	0.16	30
Trp	0.056	0.80	0.045	0.011	0.03	24

1 Values from nutrient requirements for swine, NRC 2012 (23). SID, standardized ileal digestible.

2 Proportion of total that is digested and absorbed at the ileum.

To illustrate the utility of the ideal protein computational approach, we extrapolated data in swine with the use of a small (50-kg) term-pregnant sow to estimate the likelihood of an amino acid deficiency in human mothers. This exercise is for illustration purposes only, and is not intended for direct application in human nutrition. Specifically, we interpolated the NRC swine model (23) to estimate the amino acid requirements for a 50-kg pregnant woman (**Table 2**), because tissue and accumulations in pregnant sows have been quantitated. Total maternal and conceptus gain for the final 6 wk of pregnancy was estimated at 145 g/d, with protein deposition of the late-pregnant mother (including mammary glands) being 12.5 g/d and the conceptus being 1.3 g/d. Maintenance needs were met for all amino acids if the mother consumed 680 g corn meal diet/d (as-fed basis) [Table 2; compare corn amino acids consumed (grams per day) with maintenance requirement (grams per day)]. However, a severe deficit was estimated for lysine and threonine when conceptus and maternal/mammary growth were added in (Table 2; net balance, grams per day). By these calculations, 3 other amino acids (isoleucine, valine, and tryptophan) are projected to be deficient, but to a lesser extent. Corn meal is an extreme example, but this illustrates that some alternate supplemental foods (e.g., soy legume or milk powder) would be needed to meet amino acid requirements for term-pregnant mothers. Animal nutritionists often supplement commercially available crystalline amino acids to cost-effectively meet requirements. We contend that these estimates of deficiency and the means for deriving such estimates from the composition of various ingredients and intake serve as the basis for the claim of an important gap in human nutrition knowledge.

**TABLE 2:** Use of porcine data to extrapolate daily amino acid requirements for a 50-kg term pregnant mother (including maintenance plus conceptus and mammary growth) and comparison with amounts provided by consumption of 680 g corn/d to compute net daily amino acid balance^1^

	Corn			Maintenance			Conceptus and mammary growth			Total	
Indispensable amino acid	Content,^2^ % as SID	Content, ratio:Lys	Consumed, g SID/d	SID REQT,^3^ mg/kg MBW	Amino acid, ratio:Lys	REQT, g/d	Ideal pattern,^4^ g/100 g Lys	Amino acid, ratio:Lys	REQT, g/d	Net REQT,^5^ g/d	Net balance, g/d
Lys	0.159	1.00	1.08	34.8	1.00	0.65	100	1.00	2.07	2.72	−1.64
Arg	0.271	1.70	1.85	17.1	0.49	0.32	52.0	0.52	1.07	1.40	0.45
His	0.166	1.04	1.13	12.1	0.35	0.23	34.2	0.34	0.71	0.93	0.20
Ile	0.189	1.19	1.29	29.8	0.86	0.56	58.2	0.58	1.20	1.76	−0.48
Leu	0.715	4.50	4.87	36.0	1.04	0.68	92.2	0.92	1.91	2.58	2.29
Met	0.119	0.75	0.81	9.2	0.26	0.17	27.4	0.27	0.57	0.74	0.07
Phe	0.332	2.08	2.26	25.0	0.72	0.47	56.5	0.57	1.17	1.64	0.62
Thr	0.184	1.16	1.25	44.5	1.28	0.84	83.0	0.83	1.72	2.55	−1.30
Trp	0.045	0.28	0.31	10.9	0.31	0.21	19.5	0.20	0.40	0.61	−0.30
Val	0.261	1.64	1.78	37.8	1.09	0.71	75.4	0.75	1.56	2.27	−0.49

1 For illustration purposes only, and not intended for direct application in human nutrition. MBW, metabolic body weight; REQT, requirement; SID, standardized ileal digestible.

2 Corn (International Feed No. 4–02–861, 7.2% crude protein) amino acid composition presented as SID amounts from the NRC swine model, 2012 (23). SID represents the amounts of amino acids that are absorbed and available for metabolic use.

3 Maintenance estimates taken from Tables 2–7 of the NRC swine model, 2012 (23). MBW = kg^0.75^. This REQT represents the absolute physiologic need corrected for efficiency of utilization of absorbed amino acid.

4 Ideal pattern for each amino acid relative to Lys, taken from Tables 2–8 of the NRC swine model, 2012 (23).

5 Net tissue REQT for Lys computed based on 145 g gain/d in the last 6 wk of pregnancy. Net tissue REQT for other amino acids computed by multiplying their ideal ratio to Lys × net Lysine REQT.

Under this scenario, and based on our calculations, we expect that the mammary gland growth and labile maternal protein stores needed to support early lactation would be compromised. Deficiency could impair fetal growth in young (small) sows if the deficits were severe, and infant growth could be further compromised by diminished breast-milk production. We chose not to compute lactation needs; however, it is well known that lactation more than doubles the sow’s requirement for amino acids, and it does so abruptly (22, 23). The consequences of amino acid inadequacy in lactation are the greatest in young (smaller) sows, which have the least labile body protein reserves to support lactation deficits (35, 36).

##### Global health perspective.

The basic information needed to formulate diets to meet amino acid needs in humans represents a serious gap in knowledge. The impact of this deficit must be greatest in low-income countries, in which food resources are scarce and meals are constrained by affordability. A lack of information for humans could compromise fetal growth, mammary growth, and subsequent milk output (amount and duration), as well as suckling infant growth. Collectively, stunting would occur because amino acid nutrition in late pregnancy through lactation and early growth are inextricably linked.

#### Key finding 7. Animal science research has shown that functional amino acids improve conceptus viability and prevent growth retardation of fetuses in underfed ewes and pigs.

Amino acids are at the forefront of nutrition research in preventing intrauterine growth restriction of the conceptus, which has a permanent negative impact on the growth and survival of the neonate, as well as the development of metabolic diseases and fertility in adulthood (37, 38). Some amino acids (e.g., arginine, cysteine, glutamine, and proline) are referred to as conditionally essential because under specific physiologic stages or conditions, such as pregnancy, lactation, and/or upon early weaning, they must also be provided in the diet because rates of use exceed the rate of synthesis. The development of embryos to the blastocyst stage for implantation requires leucine or arginine that initiate cell signaling via the mechanistic target of rapamycin (mTOR) to regulate protein synthesis and catabolism and induce expression of genes for IGF II, NO synthases, and ornithine decarboxylase (39). Physiologic concentrations of leucine, arginine, and glutamine stimulate activities of mTOR and ribosomal protein S6 kinase, as well as proliferation of trophectoderm cells. Cellular events associated with the elongation of ovine and porcine conceptuses during the peri-implantation period of pregnancy involve cellular hyperplasia and hypertrophy, as well as cytoskeletal reorganization during the transition of spherical blastocysts to tubular and filamentous conceptuses (40).

The concentrations of arginine, glutamine, and leucine are greater in uterine fluids than in maternal plasma, and arginine is most stimulatory to proliferation, migration, and gene expression by ovine trophectoderm cells (39, 41). Increases in uterine concentrations of these amino acids between days 10 and 16 of pregnancy coincide with the rapid growth and development of conceptuses during the peri-implantation period. Arginine is the precursor for synthesis of both NO and polyamines (putrescine, spermidine, and spermine), which are critical to the growth of the conceptus (embryo or fetus and placenta). Rates of NO and polyamine synthesis in both porcine and ovine placentae are highest during early gestation when placental growth is most rapid. Placental growth (including vascular growth) and function requires synthesis of NO and polyamines to prevent intrauterine growth restriction in both underfed and overfed dams.

The unusual abundance of arginine in porcine allantoic fluid during early pregnancy was the basis for studies to determine the effects of dietary supplementation of arginine on reproductive performance of gilts. Arginine increased the number of live-born piglets by 2/litter and litter birth-weight by 24% (39). Intravenous administration of arginine improved embryonic survival in pregnant ewes and prevented growth retardation of fetuses from underfed ewes. Intravenous administration of arginine to ewes between days 100 and 121 of gestation decreased the percentage of stillborn lambs by 23% and enhanced the birth weights of quadruplets by 23%, without affecting maternal body weight (39). Arginine as a dietary supplement for pregnant rats also increases the numbers of implantation sites and litter size by ∼3 by increasing embryonic survival (39).

### Variables affecting lactating females and suckling neonates

#### Key finding 8. Animal science research has quantified the colostrum intake required to minimize neonatal mortality and showed the essential function of early colostrum intake on gut barrier function to protect against enteric infection.

Failure of calves (42–44) or piglets (45, 46) to achieve adequate intake of colostrum is an underlying cause of the majority of deaths that occur soon after birth. Research indicates that 200 g colostrum/piglet (∼1–2 kg body weight) during the first 24 h after birth is the minimum consumption necessary to decrease the risk of mortality in weaning and to promote weight gain (47). Mammary development of the dam is important to colostrum production, but the role of specific nutrients on volume or quality is unclear. Maternal vaccination has long been recognized as important to antibody output in colostrum (48), and may be particularly beneficial in younger compared with older mothers.

Driven by the link between the volume of colostrum intake and piglet mortality, a simple method for field assessment of colostrum initiation and quantity consumed by the neonate was developed for application in pigs (termed Immunocrit) (49). This method may have field application, as a diagnostic tool, for human infant assessment. Vallet et al. (49) showed that much of the variation in early neonatal mortality in pigs (16.7%) was accounted for by inadequate colostrum intake. Among its attributes, colostrum *1*) provides a metabolic switch for glucose synthesis, *2*) promotes thermoregulation, *3*) provides the first IgG source for systemic passive immunity and gut barrier function, and *4*) promotes intestinal villi growth and/or maturation for digestion.

Protection of the intestinal barrier function is especially important. Moeser (50) summarized how various factors disrupt intestinal barrier function in piglets, including early weaning, pathogens, diet, and microbiome dysbiosis. Colostrum intake and long-term maternal nursing are especially important in preventing barrier disruption, the effects being more long-term than short-term. The importance of colostrum cannot be overstated for the piglet, but especially for the preterm infant, when the mother’s milk is not available (in premature birth or late milk secretion).

The remarkable role of the bioactive factors found in colostrum is further illustrated for reproductive tissues. The factors initiate maturation of the uterus during the first 48 h of life, which is important to long-term reproduction. This phenomenon is known as the lactocrine hypothesis. Lactocrine signaling is the transmission of bioactive factors (e.g., IGF I and relaxin) in colostrum from the mother to offspring as a consequence of nursing. Female piglets that receive colostrum have advanced onset in the development of the uterine endometrium and uterine glands that is associated with the earlier onset of expression for estrogen receptors and vascular endothelial growth factor, as well as other genes associated with differentiation of various tissues (51). Factors in colostrum also enhance testicular development in male pigs.

The practical outcomes of colostrum intake on development of neonatal reproductive tissue is nicely illustrated in an article by Vallet et al. (52), in which reproduction at the sexually mature stage of life was related to the amount of Ig derived from colostrum present in the serum (immunocrit measure) of piglets on postnatal day 1. Immunocrit measures (49) were collected in 16,762 piglets on day 1. Body weight measures were available from 15,324 (7684 male and 7640 female) piglets over a range of age from weaning to 200 d of age. Low colostrum intake was associated with decreased growth rate and increased age at puberty. Once sows reproduced, those with low colostrum intake gave birth to fewer live piglets per litter and weaned fewer piglets, and the growth rate of their subsequent litter also was reduced. The fact that colostrum consumption by newborn pigs had such long-term effects on reproduction is remarkable and emphasizes the essentiality of high amounts of colostrum intake at birth. Application for preterm human babies and for infants whose mothers produce no colostrum may consider colostrum from a surrogate mother or one of bovine origin, or perhaps a purified source of animal plasma, which also contains a vast array of bioactive factors.

#### Key finding 9. Animal science research has shown that dietary energy and amino acid nutrition during lactation can alter milk volume, and in extreme cases can alter the composition of milk.

In the food animal sector, poor milk production occurs because of genetic inferiority, physiologic dysfunction, extreme heat (which reduces feed consumption and mammary growth), and nutrient deficiency during pregnancy and (or) lactation, especially in young female animals. Unfortunately, the heat stress effects on milk volume cannot be negated by increased nutrient input (53). In domestic animals, milk volume is compromised in young dams that do not receive adequate energy and/or amino acids during lactation. A reduction in milk quality (e.g., fat and protein content) is the result of a profound nutrient deficiency. Changing the content of lactose is almost impossible because of its osmoregulatory function, and the ability to increase protein content is rather limited (unless correcting for a severe maternal dietary deficit). However, both lipid content (milk fat percentage) and quality (FA composition) are comparatively easy to alter by changing diet composition, especially in monogastric species.

The established dogma in human maternal nutrition is that the nutrient composition of breast milk is largely independent of diet, with the exception of FA composition and selected vitamins (54). In domestic swine, nutrition is the primary means of manipulating milk fat content, because FA composition is a direct reflection of dietary input (17). Despite the obvious benefits of milk protein, we know little of what regulates its concentration in milk. In the milk protein section (below), we illustrate that milk protein content can be compromised in young females by low energy and amino acid nutrition during lactation (late pregnancy and lactation nutrition are linked by mass of labile body protein reserves) and we propose that apparent increases observed in ruminant milk are possibly the result of correcting deficiencies. The rumen presents special challenges in achieving clarity regarding the effects of nutritional alternations. For example, the microbial ecosystem of the rumen extensively biohydrogenates FAs and greatly alters dietary amino acid profiles. In contrast, the effects of dietary alterations are more straightforward in monogastric swine.

##### Milk protein.

With respect to energy and high-quality protein (balanced in essential amino acids), nutritionally well-fed food animals do not exhibit an increase in normal milk protein content with further diet fortifications. Nutritional adequacy of amino acids leads to a milk protein concentration that is resistant to change. Exceptions occur that result in reductions in protein content (or protein yield), but these arise from amino acid deficiency. One example involves a dietary amino acid deficiency large enough to cause extensive loss of body protein (described below). In dairy cattle, the overfeeding of protein does not increase milk protein, but urea production increases and is reflected in milk urea secretion and compromised reproduction (55, 56).

Although milk protein content cannot be increased beyond that observed for a nutritionally well-fed state, the mouse and rat secrete free amino acids and small peptides in their milk, and these could increase amino acid output if human mammary glands function in a similar manner. Although domestic animals do not respond similarly, their mammary glands exhibit a remarkable ability to take up amino acids and to form dispensable amino acids from an array of metabolic intermediates (57, 58). In this manner, maternal tissues exhibit the propensity to compensate during short periods of nutrient deficit.

Given the relative dearth of information on amino acid requirements for human lactation, especially in young women with relatively low body protein reserves, a question arises about the extent of body protein loss that would compromise milk protein content, protein yield, or both. In the case of human mothers (especially in those who are young and have a low BMI), particularly in low-income countries, the length of time nursed and modest body protein reserves potentiate the problem and undoubtedly compromise infant growth. Data describing these important determinants of lactation are lacking for humans.

The pig has been used to study the impact of dietary energy and amino acid restriction (deficit relative to requirement for milk production) on the growth of offspring when they are completely dependent on maternal nutrients. Boyd et al. (22) predicted the effect of nutrient deprivation during lactation in young first-litter sows because milk volume and quality are most easily affected. A young sow is defined as a first-litter sow (adolescent), and typically has ∼50–60% of practical mature body weight. In these sows, Boyd et al. estimated a loss of 18% of body protein mass during a 21-d lactation with moderate restriction of an amino acid–balanced protein mixture; 45% of the loss occurred during the initial 6 d, when daily protein intake was the least. Milk secretion is known to decline as labile body protein reserves are being used, but it abruptly declines when protein reserves are depleted (36).

Touchette et al. (59) created a lysine-deficient diet for young lactating sows to study the effect of progressive increases in lysine concentration on milk secretion (indirectly estimated by litter growth). A similar approach was taken by Srichana (28). Each observed an 8–10% reduction in piglet growth rate with a deficit of >20% in the most limiting amino acid. The former reported that piglet mortality also increased as milk secretion declined. Milk protein content was not assayed in either case.

There remain 2 important points pertaining to amino acid intake and milk secretion. First, multiple studies were used to describe the linear relation between maternal amino acid intake and milk secreted (based on litter growth); litter growth increased by 2.35 times over a 21-d lactation for a 2.90-fold input (grams per day) of dietary amino acids (via protein) (22). The improved progeny growth was directly related to the increased intake of lysine and other amino acids. Second, milk protein concentration can be decreased by amino acid deficit. Clowes et al. (35) showed that milk protein content declined in relation to the amount of body protein lost; milk protein was lowest in sows that lost the most body protein. The decline in milk volume and milk protein content was further reflected by decreased piglet growth and suppressed ovarian function. Clowes et al. also determined that when amino acid needs were fully met throughout pregnancy, up to 12% body protein could be mobilized in support of a 26-d lactation without consequence to milk quantity or quality and ovarian function.

##### Milk fat.

Fat is typically the most variable component in milk, and it is the most easily manipulated by diet. Total milk fat concentration and its specific FA composition can be altered in dairy cows and sows by increasing fat and altering FA type beyond that normally fed. Dietary lipids are routinely used in the diets of lactating sows to increase total energy intake during periods of heat stress (reduced heat increment) and to provide specific FAs in support of reproduction. Rosero et al. (17) summarized the benefits of a variety of supplemental fats with the use of results from 12 studies published between 1989 and 2012, with supplemental fat ranging from 2% to 11% of the diet (as-fed basis). For each 1.0% increase in added dietary fat, total diet energy intake increased by 1.84% and milk fat output increased by 10.1 g/d. Litter growth was improved slightly (70 g/d) and maternal body weight loss was less (1 kg) over a 21-d lactation. Studies that reported milk production and milk fat content found a consistent improvement in milk yield, with increases in dietary fat, averaging 250 g/d (increase of 8% to 10%, as-fed basis), with improvements being greatest under conditions of heat stress, when the feed intake of the mother was reduced (60). Thus, milk volume and fat output can both be improved by nutrition, and total milk protein secretion was indirectly increased by improved milk volume.

Although lipid supplementation to lactating dairy cows increases milk yield, milk fat yield, and the efficiency of milk production, variation in responsiveness between cows is observed. Studies that examined the effect of individual SFAs on the production and metabolic responses of lactating cows (61–64) revealed that palmitic acid (16:0) supplementation increased milk yields and milk fat, as well as the efficiency of feed conversion to milk. Piantoni et al. (64) reported that stearic acid (18:0) increased feed intake and yields of milk and milk components, with increases being more evident in cows with higher milk yields. Greco et al. (65) showed that supplying the same quantity of FAs in the diet of dairy cows during early lactation, but altering the ratio of n–6 to n–3 FAs, influenced lactation performance and inflammatory response to an LPS challenge. In particular, reducing the ratio of n–6 to n–3 FA increased feed intake and the yield of milk and milk components.

Farm animal research makes it clear that high-quality lactation (milk volume, nutrient mass, and lactation length) is more easily achieved in mature female animals with adequate nutrient intake during lactation. Conversely, young mothers have less body mass and lower protein and fat reserves, so a deficiency in amino acids in lactation reduces milk volume and milk protein content (36), with a greater probability of milk cessation. The extra nutritional needs of young mothers represent an important knowledge gap in practical human nutrition.

In summary, milk quantity and quality are achieved through nutritionally adequate diets (especially energy and amino acids) during late pregnancy and lactation, with each of these phases being inextricably linked to body fat and labile protein reserves. Body reserves of fat and protein must be established by late pregnancy to be subsequently drawn upon in support of lactation—especially in early lactation, when energy and protein deficit is highest. Such reserves ensure high milk volume with a high nutrient content over a long period of lactation. Foundational principles involved in making transitions between physiologic states, such as pregnancy to lactation, and from early to late lactation in domestic animals were elegantly described in a classic review by Bauman and Currie (66). Although we are not aware of methods to increase milk protein content for adequately fed mothers, total milk protein output has been increased in pigs by feeding high energy diets under normal conditions, especially during heat stress. Thus, not only does fat alter the amount and nature of milk fat in domestic animals, but total protein output often increases because of greater milk volume. Conversely, milk protein content can be adversely affected by deficit amino acid intake, and this can be compounded when milk volume is reduced.

##### Global health perspective.

Epidemiologic studies indicate that considerable stunting of infant growth occurs in low-income societies during the first months of life, when children are mostly breastfed. Interventions to improve the quality and quantity of human milk are not promoted in global health, because it is assumed that even malnourished mothers are able to produce milk with adequate composition. Similar growth restriction is observed in farm animals when reductions in milk production are known to exist. Perhaps similar reductions exist in undernourished human mothers. A key question is whether maternal diets can be improved during pregnancy and in lactation to provide more and better quality milk, thereby reducing infant growth restriction, and, in extreme cases, to prevent early cessation of breastfeeding.

#### Key finding 10. Animal science research has shown that stunting of neonates occurs when they are underfed by the mother, but that growth can be recovered via formula supplementation.

In animal nutrition, the strategic use of supplemental milk replacement formula can partially compensate during periods of decreased milk production by the mother. Breast milk is the preferred source of nutrients for the neonate, because it contains many bioactive elements that support optimal growth and development. If breast milk is insufficient as a result of infection (e.g., with HIV or mastitis), agalactia, or heat stress, judicious supplementation with nutritionally balanced full-strength milk formula can reduce the stunting of growth that may otherwise occur.

Piglets consuming ad libitum milk replacement formula after consuming colostrum for 1–2 d, and independent of the sow, display growth trajectories that exceed sow-suckled controls by up to 60% (67, 68), increasing rates of protein, fat, and bone accretion. Early-life growth acceleration can be maintained into adulthood (67, 69); however, if applied too early (∼2 d of age) in a commercial farm environment (70), subsequent growth kinetics and viability falter. This might be attributable to the plethora of bioactive factors (71, 72) in sow milk that pigs weaned early onto milk replacer did not receive. Greater piglet responses to supplemental milk replacer also were observed when their dams were experiencing heat stress; formula supplementation was able to return piglet growth rates to control (nonheat-stressed) levels (73).

From a global health perspective, stunting occurs early in life, while the infant is being breastfed. Results of studies with domestic animals suggest that supplementation with milk formula during this time can recover or even accelerate growth. However, the introduction of milk formula is not fruitful under normal farming conditions, but it is an essential tool to prevent severe growth restriction, enteric damage, or death when maternal milk secretion stops or is severely reduced. Similarly, if sufficient breastmilk is unavailable for infants, judicious supplementation with a full-strength, well-balanced infant formula could be justified.

### Variables affecting offspring growth and viability during the postweaning and toddler phase

#### Extrapolating from piglets to children.

##### Estimating amino acid adequacy for an 8.0-kg child.

Herein, an analysis was performed to estimate the amino acid needs of a weaned child compared with amino acid intake in order to predict amino acid adequacy. This exercise is for illustration purposes only, and not intended for direct application in human nutrition. The analysis serves to illustrate the computational approach and estimates potential amino acid limitations that may affect the rate of growth. We used data from studies conducted on the young, weaned pig, because one cannot estimate whether a diet is adequate to sustain normal or catch-up growth in young children without more information on amino acids than we currently have for humans. Knowledge of essential amino acid requirements for growing children is very limited, and the ability to access amino acids in food ingredients is almost nonexistent; however, these data are readily available for domestic animal nutrition.

Failure to meet amino acid needs for weaned infants (voluntary or forced by milk cessation) causes profound changes in the rate and composition of growth. For example, Eisemann et al. (74) reported that progressive increases in dietary amino acids (ratio of lysine to gross energy) improved the rate and composition of growth of piglets from 1.5 to 5.5 kg body weight. Eisemann et al. observed up to a 51% improvement in the ratio of body protein to lipids. Growth rate improved by 38% (278 compared with 201 g/d), and the efficiency of food use improved by 54%. Growth occurred during an amino acid deficit (albeit less), but weight gain was composed of more fat and less protein. Because piglets were fed a constant amount of milk relative to body weight, we do not know whether the piglets would have altered their intake in order to counter the amino acid deficit.

**FIGURE 4:**
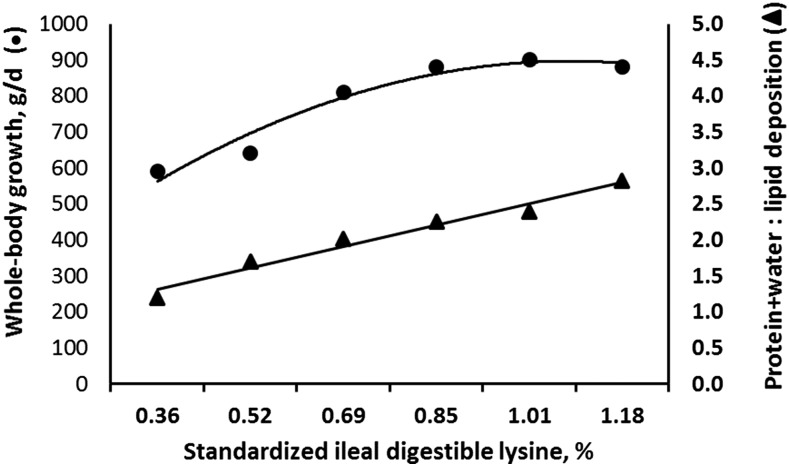
Effects of improving dietary amino acid adequacy on whole-body growth rate (upper line, left axis) and composition of weight gain (ratio of protein plus water deposition to lipid deposition; lower line, right axis) in pigs grown from 20 to 60 kg. Other essential amino acids were provided in the ideal ratio to lysine. Standardized ileal digestible lysine was computed with the use of the NRC swine data (23). Maximal growth rate was achieved only with adequate intake of available amino acids. The marginal increase in growth was almost entirely a function of lean tissue (protein and associated water). Lipid deposition rate was largely unchanged, although the percentage of lipid declined because of the relative increase in lean tissue gain. Values are means. Adapted from reference 75 with permission.

This same relation was illustrated with the use of adolescent pigs (20–60 kg) that consumed feed ad libitum in a lysine dose-response assay (75). The ratio of body protein (plus water) to lipid deposition improved 2-fold by increasing digestible lysine from 0.36% to 1.18% (as-fed basis). The whole-body growth rate improved by 50% over this same range (**Figure 4**). The fat deposition rate (grams per day) was approximately constant over this same range, suggesting that, in the absence of adequate amino acids, less growth would have occurred and the composition of growth would have been less lean (data not shown).

Furthermore, when diets deficient in amino acids are consumed ad libitum, animals may increase food intake, and this overeating results in greater lipid deposition (76). Many species (insects, mice, poultry, pigs, and humans) display this response, but it may be constrained to an upper limit of body-fat accretion (77). The conclusion is that failure to meet dietary amino acid needs can result in greater dietary energy intake in an attempt to acquire needed amino acids. The converse also appears to happen, i.e., protein is overconsumed to achieve a target energy intake.

Food animals grow more rapidly as infants and during adolescence than do their human counterparts by an order of magnitude. Consequently, the human child is expected to have an ideal amino acid requirement (pattern) that is lower and more similar in pattern to maintenance than for high rates of lean growth. Although the pattern of some amino acids is not greatly affected (the maintenance pattern being similar to the growth pattern), the relative content of threonine (in proportion to lysine) increases with more rapid growth in pigs, and this could be especially relevant for catch-up growth considerations in toddlers and infants.

A factorial analysis commonly used in animal nutrition was again used to estimate the requirement for absorbed amino acids (SID) of an 8.0-kg weaned child for maintenance plus growth (**Table 3**). The analysis considered a scenario of desired compensatory growth, considered to be 380 g whole-body gain/mo (57 g protein deposition/mo or 1.9 g protein/d). An estimated 0.34 g SID (absorbed) lysine is required per day for maintenance, but 0.55 g SID lysine/d is required to support a 380 g/mo gain composed of 15% protein. Because the magnitude of human growth is slight compared with the weaned pig, the resulting amino acid pattern more closely approximates the maintenance pattern for amino acids in pigs (Table 2).

**TABLE 3:** Use of porcine data to extrapolate daily amino acid requirements for maintenance and growth of an 8-kg child and comparison with amounts provided by consumption of 208 g corn/d to compute net daily amino acid balance^1^

	Corn			Maintenance			Growth			Total^2^	
Indispensable amino acid	Content,^3^ % as SID	Content, ratio:Lys	Consumed, g SID/d	SID REQT,^4^ mg/kg MBW	Amino acid, ratio:Lys	SID REQT, g/d	Ideal pattern,^5^ g/100 g Lys	Net REQT,^6^ g/d	SID REQT,^7^ g/d	SID REQT,^6^ g/d	Net balance, g/d
Lys	0.159	1.00	0.331	71.1	1.00	0.34	100	0.13	0.21	0.55	−0.22
Arg	0.271	1.70	0.56	38.6	0.54	0.18	90	0.12	0.15	0.34	0.23
His	0.166	1.04	0.35	25.0	0.35	0.12	45	0.06	0.07	0.19	0.16
Ile	0.189	1.19	0.39	62.3	0.88	0.30	51	0.07	0.10	0.40	−0.01
Leu	0.715	4.50	1.49	88.8	1.25	0.42	100	0.13	0.21	0.63	0.86
Met	0.119	0.75	0.25	19.7	0.28	0.09	28	0.04	0.06	0.15	0.09
Phe	0.332	2.08	0.69	64.3	0.90	0.31	52	0.07	0.14	0.44	0.25
Thr	0.184	1.16	0.38	97.3	1.37	0.46	53	0.07	0.11	0.57	−0.19
Trp	0.045	0.28	0.09	27.0	0.38	0.13	13	0.02	0.03	0.16	−0.07
Val	0.261	1.64	0.54	83.9	1.18	0.40	66	0.09	0.13	0.53	0.01

1 For illustration purposes only, and not intended for direct application in human nutrition. MBW, metabolic body weight; REQT, requirement; SID, standardized ileal digestible.

2 Maintenance plus gain.

3 Corn (International Feed No. 4–02–861, 7.2% crude protein) amino acid composition presented as SID amounts from the NRC swine model, 2012 (23). SID represents the amounts of amino acids that are absorbed and available for metabolic use.

4 Maintenance estimates taken from Tables 2–7 of the NRC swine model, 2012 (23). MBW = kg^0.75^. This REQT represents the absolute physiologic need corrected for efficiency of utilization of the absorbed amino acid.

5 Ideal pattern for each amino acid relative to Lys, taken from Tables 2–8 of the NRC swine model, 2012 (23).

6 Net tissue REQT for Lys computed based on 380 g body weight gain/mo and 15% protein in gain and 7.1 g lysine/100 g protein gain. Net tissue REQT for other amino acids computed by multiplying their ideal ratio to Lys × net Lys REQT.

7 SID REQT for all amino acids computed by dividing net REQT by efficiency of absorbed amino acid use for tissue deposition (23).

The next question is whether a particular diet would meet amino acid needs for this rate of accelerated growth (380 g/mo). To illustrate, we assumed that breastfeeding ceased, and that the 8.0-kg infant received only a corn cake diet and consumed 208 g corn/d (2.6% of body weight). This diet would not meet the maintenance requirement for lysine, threonine, or tryptophan (Table 3; net balance), resulting in weight loss, which would compound any problem of low birth weight and marginal and/or shortened lactation. Supplementation with an ingredient rich in these amino acids, such as milk powder or soy, could correct the problem. Alternatively, the corn could be supplemented directly with a mixture of the 3 crystalline amino acids. Indeed, affordable crystalline amino acid supplements are commonly used in diets for domestic livestock. When used to supplement a multi-ingredient meal and in proper dose, concern regarding any off-flavor could be minimal.

The information outlined in Table 3 could be used in designing a food mixture to support normal growth (e.g., 200 g/mo) or accelerated growth. By accelerated growth we mean the healthy growth of lean tissue as opposed to lipid accretion. Ingredient amino acid digestibility values could be approximated with the use of those determined with the pig (23), as suggested by FAO Report 92 [**Text Box 2 **, (29)]. This FAO report reflected only initial recommendations and was not yet sufficiently developed to have the specificity reflective of practical domestic animal nutrition.

Text Box 2 **FAO food and nutrition report 92, 2013**

A convened panel of experts published a report of their working group, seeking to advance the discussion of protein nutrition in humans beyond protein adequacy to amino acid adequacy. This is a notable step in bringing practical human amino acid nutriture closer to the specificity required, and is routinely used for monogastric domestic animals. This group was chaired by Paul Moughan, animal nutrition scientist from Massey University, with concepts undergirded by another animal nutrition scientist, the late Malcolm Fuller.

Key proposals included the following:

*1*) In dietary protein quality evaluation, amino acids should be treated as individual ingredients, and wherever possible, data for digestible amino acids should be reported. NRC swine SID data (23) were cited as an appropriate resource.
*2*) A new protein quality measure (digestible indispensable amino acid score) was recommended to begin the process of accounting for differences in the accessibility of individual amino acids from ingredients. The specific means to derive these estimates are illustrated in a publication from the animal nutrition laboratory of H. Stein (78).
*3*) Recommended amino acid scoring patterns (reference or ideal pattern) were suggested for infants (birth to 6 mo of age), young children (6 mo to 3 y of age) and older children (3–10 y of age).
*4*) Estimated daily amounts of amino acids required for maintenance and production were published that will allow for the evaluation of the adequacy of food combinations and amounts toward meeting requirements for early growth.

There is a sizable error in using total (rather than SID) values as previously illustrated (Table 1). We emphasize that the ideal pattern of amino acids required for marginal growth is different from that of accelerated growth. Marginal growth (low protein deposition) is heavily influenced by the pattern of amino acids required for maintenance, and is less affected by the pattern required for growth. The different patterns are shown in Table 3. For this reason, accelerated growth has slightly exacerbated needs for certain amino acids (e.g., threonine) compared with normal growth.

##### Energy considerations.

Rate of growth in weaned domestic animals (toddler phase) is a close function of the rate of protein deposition (and associated water), but adequate energy must also be consumed. Protein deposition not only requires the adequate intake of indispensable amino acids, but sufficient energy intake also is needed to support the deposition of protein. When amino acids are not deficient, protein deposition can be constrained by energy intake above maintenance. This concept was elegantly illustrated by Black and Griffiths (79) with the use of milk-fed lambs. When absorbed nitrogen was provided in excess of the requirement, nitrogen balance progressed linearly as increments of energy were consumed MJ metabolizable energy/(kg^0.75^ · d). Thus, concomitant energy intake also is especially pertinent for early correction of stunting. Children who are provided adequate and balanced amino acid intake can remain limited by inadequate energy intake to support protein deposition, thereby preventing accelerated growth.

The USDA nutrient database of human foods (80) contains good information on energy content, but it contains limited information on amino acid composition. More complete information on amino acid composition of foodstuffs can be found in the complementary database maintained by the National Animal Nutrition Program (81).

##### Global health perspective.

Recovery from early growth failure requires better information on amino acid nutrition and adequate energy intake to support growth than the human nutrition community currently has. Porcine nutrition is advanced in this regard and can inform the community with respect to the development of principles and priorities for improved nutrition of developing toddlers.

#### Key finding 11. Animal science research has shown that dietary phytase improves iron and zinc nutrition through improved bioavailability.

Phytic acid (inositol polyphosphate, phytate) is abundant in grains and legumes and acts as an antinutrient in the digestive system. Antinutrient outcomes of this substance are extensive. Phytic acid has a negative charge, which it maintains across all pH environments in the intestine, and this interferes with the digestion of positively charged proteins and the absorption of metal cations such as zinc, iron, copper, magnesium, and calcium (82, 83). Phytic acid forms stable complexes with divalent cations, with zinc ions being the most vulnerable (84), and it strongly chelates iron ions as well.

Animal scientists were the first to use dietary phytase to hydrolyze phytic acid in cereals and legumes for the purpose of releasing bound phosphorus [Lei et al. (85)]. Application of this technology evolved to using phytase at concentrations sufficient to hydrolyze >85% of dietary phytate-phosphorus, and this markedly improved iron and zinc bioavailability (86). This intervention is a clear example of animal science knowledge gained and applied that could benefit human nutrition by improving mineral utilization.

##### Zinc.

Zinc deficiency is one of the most prevalent human nutrition deficiencies worldwide (87), but, interestingly, classic work by O’Dell and Savage (88) demonstrated that no zinc deficiency was observed in chicks fed diets lacking phytic acid. With zinc deficiency being an important risk factor during pregnancy and infancy in humans (89), the potential use of supplemental phytase is even more compelling, especially in developing countries. Endogenous zinc is secreted into the duodenum via the pancreas in quantities that are 2–4 times greater than in dietary consumption. In order to sustain systemic zinc status, much of the secreted zinc must be reabsorbed. Accordingly, dietary phytic acid may complex up to 65–70% of endogenous zinc, in addition to 30–35% of the zinc contained in feed ingredients.

The consequences of zinc deficiency are more complex than for iron, but they are less overtly expressed. Zinc is catalytically and structurally involved in >300 enzymes. In domestic animal nutrition, the risk of zinc deficiency is very low because of dietary fortification, and it is even lower with the use of phytase supplementation. Maladies arising from moderate zinc deficiency in young children include growth retardation, reduced appetite, failure to thrive, impaired immune function, delayed neurological and behavioral development, and increased susceptibility to infectious disease such as diarrhea, pneumonia, and malaria (90). Groups that are especially at risk include preterm and low-birth-weight infants, children from pregnant and lactating mothers who are deficient in zinc (especially young adolescent mothers), older breastfed infants and toddlers without access to zinc-rich complementary foods, and young children consuming grains and legumes that are typically high in phytic acid. The means to avert the latter has come to light recently through domestic animal research with the use of dietary phytase (86).

##### Iron.

**FIGURE 5:**
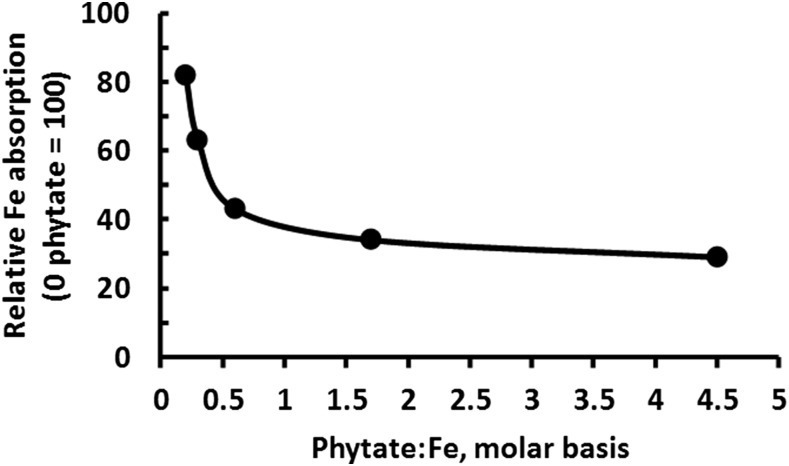
Impact of phytate concentration in digesta relative to iron content on iron absorption by adult humans. The 4.0–4.5 phytate:Fe molar ratio reflects the diet of a weaned pig with 200 ppm added iron (sulfate form). At this ratio, the relative rate of iron absorption is only ∼30%. Reduction of the phytate:Fe molar ratio to 0.5 (∼50% absorption) is similar to a diet for weaned pigs receiving iron fortification (200 ppm), but with 2000 phytase units/kg diet. Values are means. ppm, parts per million. Adapted from reference 83 with permission.

In developing countries, ∼50% of pregnant woman and 40% of preschool children are estimated to be anemic (91). Again, the consumption of cereal grains and legumes is problematic because phytic acid chelates a large percentage of dietary iron. A powerful illustration of how phytate destruction improves iron absorption in humans was provided by Hurrell (83) (**Figure 5**). The typical diet of the weaned pig has a phytate-to-iron molar ratio between 4.0 and 4.5, but the growing use of phytase concentrations that destroy 85% of phytate yield an approximate phytate-to-iron ratio of 0.50, which would increase iron absorption by 55% (Figure 5).

In countries in which the prevalence of anemia is high, use of iron-fortified foods and oral iron supplementation are recommended nutritional interventions (92). Because phytate is a potent chelation molecule (93, 94), phytase degradation of phytic acid should be considered to be an allied intervention strategy. Phytate destruction is becoming a strategic priority for improving iron uptake in animal nutrition, with the result of a greater response, even though less supplemental iron is provided. Accordingly, dietary phytase supplementation has been extensively adopted in poultry and swine nutrition.

A practical illustration of using dietary phytase to virtually eliminate phytic acid interference and to improve iron absorption was provided by Stahl et al. (95) with weaned pigs. In their study, the addition of phytase (1200 phytase units/kg diet) improved hemoglobin concentration by 24% in young anemic pigs over control pigs, each fed a high–phytic acid basal diet without supplemental iron (1.34% phytic acid; 52 parts per million ingredient iron). In addition, hemoglobin concentrations were similar to pigs fed a diet fortified with 50 parts per million iron (FeSO_4_ · 7H_2_O), suggesting that phytase improved iron bioavailability from the diet, and it also may have improved iron reabsorption.

Furthermore, dietary phytase supplementation has resulted in improvements in growth exceeding those that can be explained by mineral release alone. For example, in a study with weaned pigs supplemented with 2500 phytase units/kg diet, growth was further improved (5.7%) in diets expected to be nutritionally adequate (96); stool firmness likewise improved with phytase addition. In a second study, a phytase source was used that is more capable of complete dephosphorylation to inositol. Growth was improved by 9.1%, which primarily was due to improved food utilization (7.0%). The basis for this unanticipated effect is unclear, but ≥1 of the following may be involved: improved iron absorption, inositol release, or amino acid release. Phytic acid inhibits protein solubility (97) and increases endogenous mucin amino acid loss (98).

A concern that improved iron availability might promote *Escherichia coli* proliferation is warranted. Tightly bound iron is believed to make it unavailable to many strains of *E. coli* and serves to nutritionally deprive them (99). The low content of iron in milk and the withholding of iron by milk-binding proteins may be a physiologic attempt to restrict iron to pathogenic *E. coli*. In this context, increasing unbound iron in the digesta seems counterintuitive if it leads to increased incidence of diarrhea. Although intestinal health should remain a concern, weaned pig stool scores either improved with phytase supplementation or were unaffected, and treatment for digestive pathogens was not increased (96). These tests were conducted under the supervision of licensed veterinarians, who set the standard for stool evaluation and medical treatment. Also, iron was not being released as a bolus, the total tract amount of iron in the entire length of the weaned pig intestinal tract at any point in time would have been 30–40 mg (assuming 260 mg Fe/kg diet and a 115-g diet consumed every 6 h), and absorption would have been competing with pathogens for the increase in available iron. We therefore infer that an intervention with dietary phytase could be a means of improving iron status in human infants without supplementing dietary iron.

##### Global health perspective.

Global nutrition initiatives have focused on supplementing children and mothers with iron and zinc, rather than improving the absorption of these elements that are present in the diet. There is no zinc deficiency in domestic animals in the absence of phytic acid (88, 100), which suggests that phytic acid destruction is an important intervention for improving trace mineral bioavailability in nonfortified diets. Domestic animal studies also have shown improved iron status in weaned pigs fed phytase. Destruction of the antinutrient phytic acid by the dietary microaddition of phytase is an intervention that deserves consideration by the global nutrition community.

#### Key finding 12. Animal science research has identified prospective interventions to mitigate postweaning environmental enteropathy.

Animal husbandry may offer relevant lessons related to optimizing the growth of children in the developing world. In particular, pigs and humans share very similar intestinal physiology, and pigs have been studied extensively to develop nutritional strategies to enhance intestinal health and growth. Applications may find value in reducing environmental enteropathy, a chronic, subclinical intestinal malady in stunted children exposed to poor sanitation and fecal pathogens.

Optimum nutrition protocols for managing piglets postweaning is a major focus in swine production, because postweaning is a period of increased diarrhea and intestinal dysfunction leading to failure-to-thrive syndrome (stunting) in piglets. For years this intestinal dysfunction has been managed in the industry with the use of antibiotic growth promoters to dampen the effects of transitioning piglets through the stress of weaning. However, with the feed directives associated with the removal of antibiotics as growth promoters in 2017, the animal industry needs to find alternatives to feeding antibiotics. Some of the nutritional interventions under evaluation in the swine industry may cross over to enhance human health and will be discussed below.

##### Lipids.

Medium-chain FAs are saturated 6- to 12-carbon FAs that are natural components of TGs in milk fat and some oils (coconut, palm, and cuphea seed oils). Medium-chain TGs have been known to exhibit bactericidal effects since the 1950s (101). In addition, medium-chain FAs and TGs have specific nutritional and metabolic effects, including rapid digestion, passive absorption, and obligatory oxidation, making them particularly interesting in the nutrition of young animals (102). Feeding weaning piglets medium-chain TGs inhibited microbial counts within the intestine (103–105) and improved growth performance (104, 106).

##### Protein and amino acids.

Dietary proteins elicit a wide array of nutritional and biological functions. The source and concentration of dietary protein are known to influence enteric health in piglets (107). Dietary proteins and individual amino acids play critical physiologic regulatory roles throughout the body, but especially in the intestine, beyond their traditional role in protein synthesis. Physicochemical properties, amino acid concentration, and bioactive peptides affect mechanical, hormonal, and neuroendocrine functions of the gastrointestinal tract (108, 109). In-depth reviews on this topic in pigs have been published by Wu (110, 111).

Select amino acids also have unique roles in gut development, function, and health. l-glutamine is a prime example (112). Briefly, dietary l-glutamine (10 g/kg diet) provided in weanling pig diets for 7 d increased body-weight gain and altered the expression of genes involved in the morphologic and physiologic properties integral to enhancing gut health. Notably, Wu and coworkers (112) observed an increased expression of genes involved in energy supply to enterocytes, protein synthesis, and cellular antioxidant capacity, and a decreased expression of genes promoting oxidative stress and immune activation (113). Additional studies supplementing 20 g l-glutamine/kg diet to weaning pigs demonstrated an increased mean daily gain and higher feed intake while reducing the incidence of diarrhea from 21 to 35 d of age (114). Other mechanistic measures included an increased intestinal mass and villus height-to–crypt depth ratio associated with increased mTOR signaling in the enterocyte (114, 115). In addition, precursors of l-glutamine (*N*-carbamylglutamate and glycyl-glutamine) have the same protective roles as l-glutamine in intestinal health and increased growth in weanling pigs (116, 117). Collectively, l-glutamine supplementation during intestinal stress may have beneficial effects on human environmental enteropathy, and there seems to be a wide range of dose effectivity.

Weanling pig diets supplemented with l-arginine are implicated in the increased maintenance of intestinal morphology. Most work with arginine indicates a role for increased protein synthesis in the intestine, increased surface area by increasing villus height and crypt depth, and decreased accumulation of NO in the gut (118). Suckling piglet studies show benefits from dietary-supplemented arginine and argue that arginine is an essential amino acid during the suckling phase because milk is a poor source of arginine and precursors (119–121). Zhu et al. (122) demonstrated that postweaning arginine supplementation in pig diets enhanced intestinal integrity and mucosal barrier function. Postweaning arginine synthesis involves multiple organs, and although feed typically contains sufficient arginine for metabolic processes, during stress or disease, arginine becomes essential to protect the intestine by increasing numbers of intraepithelial lymphocytes, mast cell number, and decreasing apoptosis in the Peyer’s patches of LPS-challenged pigs (122). However, unlike with glutamine, defining the pharmacologic dose of dietary arginine is complex. Piglet studies suggest different biological outcomes, depending on the stage of growth and the dose of arginine supplied; low doses have relatively no effect, whereas high doses that enhance intestinal function may have adverse effects on the microvascular system (118).

Plasma-derived protein concentrates (PPCs) are commonly used in the diets of weanling pigs to mitigate intestinal inflammation and encourage feed consumption. The addition of PPCs to diets provides a source of high-quality protein and Ig. Torrallardona (123) reviewed 43 studies in which PPCs were fed to weanling pigs. Studies consistently showed increased feed intake and weight gain in weanling pigs fed PPC products. In addition, protein conversion efficiency in PPC-fed animals was significantly higher than it was in soy protein–fed controls, and the protein was converted to lean growth because fat mass was not affected by diet (124). Many studies have indicated that the use of plasma protein provides not only increased growth in piglets, but that these animals have improved immune status because of the increased percentage of Ig in the plasma protein (123). Finally, plasma protein can be processed 2 different ways to produce 2 separate final products: the PPCs consisting of 80% protein on a weight basis with >15% Ig included in this protein source, or serum-derived bovine Ig (containing 92% protein and >50% IgG), which is already being used to treat human clinical enteropathy under the supervision of a physician (125). Taken together, protein sources collected from plasma or serum provide a concentrated source of essential amino acids involved in the maintenance of intestinal growth and support the mucosal immune system by providing a dietary source of IgG.

Supplemental dietary lactoferrin (porcine or bovine) or derivatives of lactoferrin have also been examined in weanling pig studies, and have been demonstrated to have antimicrobial properties and result in reduced postweaning diarrhea, leading to increased growth performance (126, 127). More recently, a piglet model of protein and energy malnutrition (128) provided evidence that cow milk and, to a greater extent, cow milk with high lactoferrin (via transgenic overexpression of human lactoferrin to mimic the concentration found in human milk) improved growth rate and intestinal morphology and function.

In summary, the specific amino acids, high-quality protein sources with Ig, and bioactive proteins produced in milk have the potential to be used as nutritional interventions to help improve the growth and development of children in developing countries. These supplements could have important roles in improving intestinal morphology and function for nutrient utilization, as well as in improving immune function in children suffering from environmental enteropathy.

##### Minerals.

The zinc requirement for weanling pigs is 100 mg/kg diet (NRC, 2012) (23); however, concentrations up to 3000 mg/kg are used as an effective tool in reducing or preventing postweaning diarrhea in piglets (129). The mode of action of zinc in reducing the effects of weaning stress and enhancing intestinal health are multifactorial. Studies of weanling pigs have shown that high concentrations of dietary zinc increase growth, reduce diarrhea, decrease intestinal mast cells (histamine release), reduce chloride secretion and intestinal permeability, and prevent *E. coli* adhesion (130–133). These data suggest multiple physiologic functions of zinc that contribute to the overall protective effects in piglet intestinal health.

##### Global health perspective.

Environmental enteropathy is a subclinical condition with major implications for failure to thrive in neonates through the first 1000 d of life that can have lifelong implications, even shortening the life expectancy of individuals in low- and middle-income countries (134). Environmental enteropathy occurs in inhabitants exposed to poor sanitation and hygiene, which leads to chronic exposure to fecal pathogens. Such exposure causes structural and inflammatory changes in the small bowel, ultimately reducing its function. Hallmarks of the condition are increased intestinal permeability, impaired gut immune function, malabsorption, growth faltering, and potentially oral vaccine failure, all in seemingly asymptomatic individuals without overt diarrhea (135). Animal science research shows that several specific nutrients, including lipids, proteins and amino acids, and zinc can effectively abrogate environmental enteropathy.

## Conclusions

We have identified 12 important innovations and knowledge gaps with the potential to positively transform the outcomes of pregnancy and maternal and child health. Whether or not interventions derived from these findings become part of nutritional recommendations for global maternal and child health depends on further research on their efficacy, effectiveness, cost, and acceptability, as well as the education of citizens and health care providers throughout the world.

We have provided evidence that domestic farm animals are fed diets that are more nutritionally adequate than those consumed by pregnant and lactating human mothers and infants in low-income countries. Animal nutritionists also feed younger, smaller mothers lactation diets that are more highly fortified than are diets for older female animals because young females have lower body reserves to buffer dietary inadequacies and to prevent a decline in milk production. Our use of a pig model to probe amino acid adequacy for 2 lifecycle stages for humans and the presentation of what, to our knowledge, are novel approaches to cereal-based foods (antinutrient enzymes) provide an informed basis for identifying priorities for applied human nutrition studies intended to help improve maternal and infant health in low-income countries within the global nutrition community.

Overall, the purpose of this review was to catalyze thinking and discussion about potential interventions that may be worthy of further consideration. More broadly, we believe that better appreciation of the close linkage between medicine and agriculture will identify opportunities that will enable faster and more efficient innovations in global maternal and infant health. After all, the goal of agriculture is to produce a nutritious, safe, abundant, and affordable supply of food that is permissive to the health of humans and animals.

## Footnotes

11 Abbreviations used: EFA, essential FA; IGF, insulin-like growth factor; mTOR, mechanistic target of rapamycin; PPC, plasma-derived protein concentrate; SID, standardized ileal digestible.
